# 

**Published:** 1902-01

**Authors:** 


### Society Notes.

### Health Officers' Association of Texas.

The fourth semi-annual meeting of the Health Officers' Association of Texas will convene at Austin, Tuesday, January 14, 1901. The indications are that we will have a large and enthusiastic meeting. All health officers, city and county, and those engaged in sanitary work are eligible for membership.

J. E. Wilson, M. D., Secretary,
327 Main St., Dallas, Texas.

